# HfO_x_/AlO_y_ Superlattice‐Like Memristive Synapse

**DOI:** 10.1002/advs.202201446

**Published:** 2022-05-29

**Authors:** Chengxu Wang, Ge‐Qi Mao, Menghua Huang, Enming Huang, Zichong Zhang, Junhui Yuan, Weiming Cheng, Kan‐Hao Xue, Xingsheng Wang, Xiangshui Miao

**Affiliations:** ^1^ School of Optical and Electronic Information and School of Integrated Circuits and Wuhan National Laboratory for Optoelectronics Huazhong University of Science and Technology Wuhan 430074 P. R. China; ^2^ Hubei Yangtze Memory Laboratories Wuhan 430205 P. R. China

**Keywords:** analog switching, conductive filaments, memristive synaptic device, neuromorphic computing, superlattice‐like

## Abstract

The adjustable conductance of a two‐terminal memristor in a crossbar array can facilitate vector‐matrix multiplication in one step, making the memristor a promising synapse for efficiently implementing neuromorphic computing. To achieve controllable and gradual switching of multi‐level conductance, important for neuromorphic computing, a theoretical design of a superlattice‐like (SLL) structure switching layer for the multi‐level memristor is proposed and validated, refining the growth of conductive filaments (CFs) and preventing CFs from the abrupt formation and rupture. Ti/(HfO_x_/AlO_y_)_SLL_/TiN memristors are shown with transmission electron microscopy , X‐ray photoelectron spectroscopy , and ab initio calculation findings corroborate the SLL structure of HfO_x_/AlO_y_ film. The optimized SLL memristor achieves outstanding conductance modulation performance with linearly synaptic weight update (nonlinear factor *α* = 1.06), and the convolutional neural network based on the SLL memristive synapse improves the handwritten digit recognition accuracy to 94.95%. Meanwhile, this improved synaptic device has a fast operating speed (30 ns), a long data retention time (≥ 10^4^ s at 85 ℃), scalability, and CMOS process compatibility. Finally, its physical nature is explored and the CF evolution process is characterized using nudged elastic band calculations and the conduction mechanism fitting. In this work, as an example the HfO_x_/AlO_y_ SLL memristor provides a design viewpoint and optimization strategy for neuromorphic computing.

## Graphical abstract

A superlattice‐like (SLL) switching layer design is presented to achieve a high‐performance analog‐type memristive synapse for neuromorphic computing. HfO_x_/AlO_y_ SLL memristors exhibit high conductance modulation linearity, fast operation speed, and long‐term data retention. AlO_y_ layers are set as migration barriers of oxygen vacancy periodically to enhance the controllability of oxygen vacancy and avoid the abrupt formation/rupture of conductive filaments.

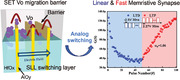

## 1 Introduction

Brain‐inspired neuromorphic computing is regarded to be a promising computation architecture for breaking the von Neumann bottleneck,^[^ ^1^ ^]^ and has been used for artificial intelligence ,^[^ ^2^, ^3^ ^]^ demonstrating significant advantages in pattern recognition applications and even surpassing human‐level performance in some cases.^[^ ^4^ ^]^ Neurons are computing units responsible for integrating incoming spikes and generating a fire signal when a specific threshold is met in hardware‐based neuromorphic computing. Because synapses link neurons and distribute the signals weighted by the synaptic strength, hardware‐based synapses must store the connection weights and conduct matrix multiplication. A neuromorphic computer system contains a large number of synaptic devices, which outnumber neuron devices and take up the majority of the chip area.^[^ ^5^ ^]^ As a result, creating low‐power and small‐size electronic synapses can effectively lower the power consumption and area of neuromorphic computing circuits. Because of its simple construction, low power consumption, scalability, and process compatibility, memristive synaptic devices owning gradual conductance adjustment capabilities are viewed as the more attractive alternatives for synaptic devices than complementary metal‐oxide‐semiconductor transistor (CMOS) circuit based electronic synapses.^[^ ^6^, ^7^ ^]^ An ideal synaptic memristor in neuromorphic computing chips should have a wide‐range conductance, long‐term retention, low power consumption, high speed, high endurance, high uniformity, and so on. Furthermore, Kirchhoff's law states that a memristor crossbar array can perform vector‐matrix multiplication of voltage input vector and conductance matrix itself in a single step.^[^ ^7^, ^8^ ^]^ It is revealed, in particular, that the linearity and symmetry of conductance modulation in memristive synaptic devices are two key characteristics improving the accuracy of a neuromorphic computing system.^[^ ^9^, ^10^ ^]^

The key prerequisite for memristors to be employed as efficient synaptic devices is that they have analog switching behavior rather than the binary switching process.^[^ ^6^ ^]^ Filamentary memrsitors, on the other hand, are never easy to achieve analog switching behavior because its conductive filament (CF) usually develops or breaks suddenly.^[^ ^11^, ^12^, ^13^ ^]^ Many techniques to increase the synaptic performance of memristors have been presented in the recent years, including improving the operation method and managing the formation/rupture process of CF. Although refining the operation approach, such as raising/reducing the amplitude or breadth of stimulus pulses, can partially eliminate some non‐ideal elements,^[^ ^14^, ^15^ ^]^ this leads to more sophisticated peripheral circuit design. As a result, the device must be redesigned and optimized, as well as the ability of memristors to adjust the CF formation/rupture process. Wu et al. designed an electro‐thermal modulation layer in a HfO_x_‐based memristor and used numerous week CFs to produce a linear analog switching mechanism.^[^ ^16^ ^]^ By changing the size of the CF, Woo et al. established a linear conductance modulation in AlO_x_/HfO_2_ bilayer structure memristor.^[^ ^17^ ^]^ Jiang et al. demonstrated an analog switching mechanism in a Ta/HfO_2_ memristor via channel composition modulation.^[^ ^18^ ^]^ However, there are still concerns with data preservation, speed, and linearity with these methods.

**Figure 1:**
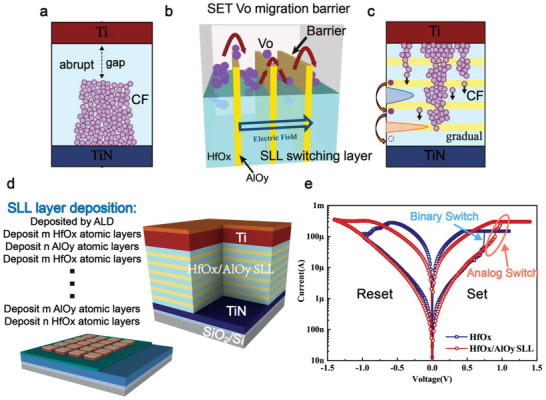
Superlattice‐like switching layer design. a) Conductive filament (CF) schematic of Ti/HfO_x_/TiN memristor, which is a typical binary switching device with a robust CF. b) Design conceptsuperlatticettic‐like (SLL) switching layer, where the AlO_y_ layers act as barriers to hinder the transmission of oxygen atoms. c) Preconceived CFs morphology of Ti/(HfO_x_/AlO_y_)_SLL_/TiN memristor fabricated following the design inspiration, with the insert of several AlO_y_ binary layers in the HfO_x_ switching layer, the oxygen vacancy (V_O_) CF will be weakened and the formation/rupture process will be gradual because, during the migration process, the oxygen ions have to overcome the barrier continually. d)Test structure schematic and cross‐sectional schematic of the HfO_x_/AlO_y_ SLL memristor, the size of one cell area is 100 µm×100 µm. Inset is the deposition method of HfO_x_/AlO_y_ SLL layer by atomic layer deposition (ALD). e) Comparison of direct current (DC) current‐voltage (*I–V*) characteristics of HfO_x_/AlO_y_ SLL memristor and HfO_x_ memristor, realizing the transform from binary to analog at both SET and RESET process by utilizing the HfO_x_/AlO_y_ SLL switching layer.

In this paper, we present a superlattice‐like (SLL) switching layer design to achieve a high‐performance analog‐type memristor with high conductance modulation linearity, fast operation speed, long‐term data retention, and CMOS process compatibility. Binary‐type memristors are usually associated with the sudden formation/rupture of a robust CF, according to earlier study.^[^ ^6^ ^]^ **Figure** **1**a describes the CF schematic of Ti/HfO_x_/TiN memristor (the complete evolutionary process of CF is shown in Figure S2, Supporting Information), illustrating a binary switching mechanism with or without the presence of oxygen vacancy (*V*_o_) channel inside the oxide.^[^ ^19^ ^]^ Thus, the key to achieving the analog‐type memristor is how to avoid the abrupt formation/rupture of CF. The superlattice structure, which may influence electron migration in the conduction band via the short‐period combination of multilayer heterojunctions,^[^ ^20^ ^]^ gives us inspiration, Similarly, by utilizing the different migration barrier of oxygen ion in the other metal‐oxide film, for example, Al_2_O_3_, we can design a superlattice‐like functional structure by periodical barrier‐layers in the migration path of V_O_ in the switching layer to gracefully control the CF, that is (HfO_x_/AlO_y_)_SLL_. As demonstrated in Figure 1b, it would generate a gradual barrier overcoming characteristic for analog‐type memristors under consecutive pulses, similar to hurdling.

## 2 Results

### 2.1 SLL Memristor Design and Fabrication

Following the above inspiration, we fabricated a Ti/(HfO_x_/AlO_y_)_SLL_/TiN SLL memristor, with HfO_x_‐based memristor selected as basic device because of its good memory performance (high‐speed operation, big ON/OFF ratio, reliable switching endurance, scalability, and high device yield).^[^ ^21^ ^]^ After comparing the *V*_O_ formation energy of six common binary metal oxide memristive materials (Al_2_O_3_,^[^ ^22^ ^]^ HfO_2_,^[^ ^13^ ^]^ *γ*‐Ta_2_O_5_,^[^ ^23^ ^]^ TiO_2_,^[^ ^24^ ^]^ ZnO,^[^ ^25^ ^]^ and ZrO_2_^[^ ^26^ ^]^) presented in Figure S4, Supporting Information, we chose AlO_y_ film as the barrier layer based on its highest *V*_O_ formation energy, process maturity, and compatibility. AlO_y_ film is typically introduced into the interface between HfO_x_ and TiN to promote uniformity^[^ ^27^ ^]^ or utilized as dopants to improve the retention of HfO_x_ memristors due to its higher bond energy.^[^ ^28^ ^]^ In our design, by inserting several AlO_y_ barrier layers in the HfO_x_ switching layer, the *V*_O_ CF will be weakened and the formation/rupture process is transformed to be gradual, because during the migration process, the oxygen ions must continuously overcome the barrier continually under the consecutive pulses of limited energy, as shown in Figure 1c.

Figure 1d shows a schematic representation of a typical HfO_x_/AlO_y_ SLL memristor device. The HfO_x_/AlO_y_ SLL memristor test sample is composed of Pt (10 nm)/Ti (50 nm)/(HfO_x_/AlO_y_)_SLL_/TiN (100 nm) which was fabricated on the SiO_2_/Si substrate. Atomic layer deposition (ALD) technology is particularly well suited for the production of the HfO_x_/AlO_y_ SLL layer, because of its ability of self‐limiting atomic layer growth. As indicated in the inset figure of Figure 1d, HfO_x_ and AlO_y_ were alternately deposited in atomic layer form by ALD at 250 ℃, first depositing *m* atomic layers of HfO_x_, then *n* atomic layers of AlO_y_, and repeating the above procedure for several cycles. Meanwhile, using the same process conditions, a Pt (10 nm)/Ti (50 nm)/HfO_x _(50 HfO_x_ cycles)/TiN (100 nm) control sample was fabricated. Figure 1e shows the *I*–*V* characteristics of the HfO_x_/AlO_y_ SLL memristor and the HfO_x_ control sample. By utilizing the HfO_x_/AlO_y_ SLL switching layer, the switching behavior of HfO_x_‐based memristor was indeed transformed from binary to analog during both SET and RESET processes. Meanwhile, the resistance states and operating voltage of SLL devices are larger than HfO_x_ memristors due to the presence of AlOy barrier layers.

### 2.2 Superlattice‐Like Film Characterization

**Figure 2:**
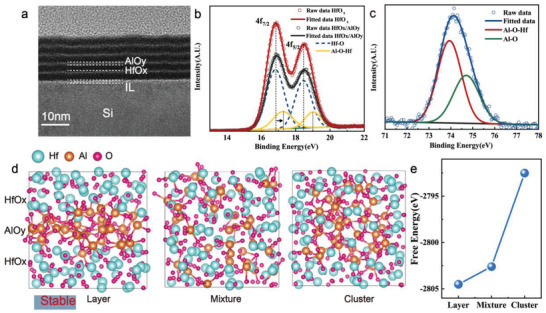
Analysis of HfO_x_/AlO_y_ SLL film. a) High‐resolution transmission electron microscopy (HR‐TEM) of 15 nm HfO_x_/AlO_y_ (30 cycles/10 cycles) SLL film with clear interfaces between HfO_x_ layer and AlO_y_ layer. X‐ray photoelectron spectroscopy (XPS) of peaks for b) Hf 4f and c) Al 2p of HfO_x_/AlO_y_ SLL film and HfOx film. The Hf‐O peak consists of a unique 4*f*_7/2_‐4*f*_5/2_ component at a 16.9 eV binding energy with a spin‐orbit with a splitting value of 1.6 eV in HfO_x_ film. The Hf 4f peak of HfO_x_/AlO_y_ SLL film shift slightly upward about 0.4–0.5eV, indicating a mixed structure with Al‐O‐Hf bonding was formed in the film. The peak of Al 2p can be deconvoluted into two peaks corresponding to Al‐O‐Hf and Al‐O at 73.9 and 74.7 eV, respectively. Before measurement, a 1 nm surface layer was removed to avoid contamination. g) Different amorphous atomic structures (layer, mixture, and cluster) of the relaxed HfO_x_/AlO_y_ film. e) Free energy of three different atomic structures, where the layer is the most stable structure with the lowest free energy.

The microscopic structure of HfO_x_/AlO_y_ SLL film is characterized by the high‐resolution transmission electron microscopy (HR‐TEM) in **Figure** **2**a and the element distribution is shown in Figure S7, Supporting Information. At the same process conditions as the above‐mentioned memristor, HfO_x_/AlO_y_ (each 30 cycles/10 cycles) SLL film with five periodical cycles is deposited on a flat single‐crystal Si substrate (chemical mechanical polishing processed). Despite the fact that HfO_x_ and AlO_y_ are both amorphous and disorderly within layers, the atomic layer distribution is macroscopically organized as multilayer (perpendicular to the interface) in the vertical direction, and the HfO_x_ layers and AlO_y_ layers can be clearly distinguished with obvious interfaces. The initial resistance and forming voltage (*V*_F_) of the SLL memristor will become excessively high as the thickness of AlO_y_ barrier layers increases, and the *V*_F_ close to 5 V is detrimental for memristor integration with transistors. Meanwhile, as shown in Figure S5, Supporting Information, the high *V*_F_ also increases the risk of thermal breakdown during the forming process.

As a result, in the following, we optimized the thickness of the HfO_x_ and AlO_y_ layers, as well as the number of periods. We used the first‐principles calculation to guide the optimization, and carried out density functional theory (DFT) calculation using the plane‐wave basis set and the projector augmented‐wave method as implemented in the Vienna Ab initio Simulation Package (VASP).^[^ ^29^, ^30^ ^]^ As shown in Figure 2d, we established three amorphous HfO_x_/AlO_y_ models with different structures, including layer, mixture, and cluster, and then we calculated the system free energy after relaxation of different structures and the results are shown in Figure 2e, the calculation method is described in the Experimental Section. In comparison to the other two structures, the layer structure has the lowest free energy, indicating that in the HfO_x_/AlO_y_ multilayer film, HfO_x_ and AlO_y_ exist as atomic layers are more stable than HfO_x_ and AlO_y_ exist as a mixture or AlO_y_ cluster. According to the theoretical support, we can reduce the thickness of the AlO_y_ layers to minize the *V*_F_ and increase the number of cycles to improve the number of conductance states, without fear of damaging the SLL structure. Figure 2b,c shows the Hf 4f and Al 2p X‐ray photoelectron spectroscopy (XPS) spectra of a HfO_x_/AlO_y_ (each 3 cycles/1 cycles) SLL film with 13 periodical cycles and a HfO_x_ film. As shown in Figure 2b, in HfOx film, only the Hf‐O peak consisting of a unique 4*f*_7/2_‐4*f*_5/2_ component at a binding energy of 16.9 eV and a spin‐orbit with a splitting value of 1.6 eV was observed. However, the Hf 4f peak of the HfO_x_/AlO_y_ SLL film shifts slightly upwards, and an optimum fit necessitates an additional component with a 0.4–0.5 eV shift to higher binding energy, indicating that the film formed a mixed structure with Al‐O‐Hf bonding.^[^ ^31^, ^32^ ^]^ Figure 2c depicts the Al 2p peak of the HfO_x_/AlO_y_ SLL film, which can be deconvoluted into two peaks at 73.9 and 74.7 eV, corresponding to Al‐O‐Hf and Al‐O, respectively.^[^ ^29^, ^30^, ^31^ ^]^ These findings imply that in the HfO_x_/AlO_y_ (each 3 cycles/1 cycles) SLL film, HfO_x_ and AlO_y_ are still not entirely mixed as existing as atomic layers, and that Al‐O‐Hf bonds are formed at the interfaces.

### 2.3 DC Performance

**Figure 3:**
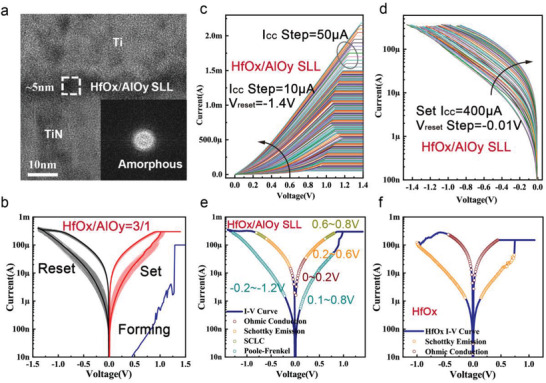
DC *I–V* characteristics of the SLL memristor where the SLL film is composed of 13 periodical HfO_x_/AlO_y_ (each 3 cycles/1 cycle). a) HR‐TEM image of the HfO_x_/AlO_y_ SLL memristor, and the insert image is corresponding fast Fourier transform (FFT image) of the HfO_x_/AlO_y_ SLL layer. b) DC *I–V* characteristics of the HfO_x_/AlO_y_ SLL memristor under ±1.4 V sweeping voltage with a SET compliance current (*I_CC_*) of 300 µA. The sweeping voltage range of forming process is 0–1.5 V and with an *I*_CC_ of 100 µA. c,d) Gradual SET and Reset process of HfO_x_/AlO_y_ SLL memristor. Gradual SET by increasing *I*_CC_ from 50 µA to 1.5 mA with a step of 10 µA and from 1.5 to 2.2 mA with a step of 50 µA. Gradual RESET by increasing the sweep stop voltage from −0.85 to −1.47 V with a step of −0.01 V. e) Fitting of the conductance mechanism for the analog switching process. For low resistance state (LRS), with the increase of applied voltage, the conduction of electrons experienced Ohmic conduction (0–0.2 V/0–−0.1 V), Schottky emission conduction (0.2–0.6 V/−0.1–0.6 V) and space charge limit current (SCLC) (0.6–0.8 V/−0.6–−0.8 V), respectively. For the high resistance state (HRS), the device follows Poole‐Frenkel (PF) emission (0.1–0.8 V/−0.2–−1.2 V). f) Conduction mechanisms of HfO_x_ memristor during the binary switching process. At LRS, the device follows Ohmic conduction and follows Schottky emission conduction at HRS.

According to the optimization results (details of the optimization process are provided in Figure S6, Supporting Information), decreasing the thickness of AlO_y_ layers can reduce the *V*_F,_ while increasing the number of cycles can increase the number of conductance states, which are consistent with our design expectation. The memristor performs best in analog switching when the SLL switching layer is constructed of 13 cycles of three atomic layers of HfO_x_ and one atomic layer of AlO_y_. Table S1, Supporting Information shows the atomic concentration of the HfO_x_/AlO_y_ (3:1) SLL film, because to the lower deposition temperature, Hf and Al are not fully oxidized, resulting in the creation of non‐stoichiometric HfO_x_ and AlO_y_. **Figure** **3**a displays a cross‐sectional HR‐TEM image of a HfO_x_/AlO_y_ (3:1) SLL memristor with a 5 nm SLL layer, and the fast Fourier transform (FFT) inset image clearly shows that the SLL layer is amorphous. Figure 3b demonstrates the 100 consecutive direct current (DC) current‐voltage (*I*–*V*) characteristics of the HfO_x_/AlO_y_ (3:1) SLL memristor with a compliance current (*I*_CC_) of 300 µA in SET process and a stop voltage of −1.4 V in RESET process, and a switching window of ≈10 can be obtained. The initial resistance is six orders of magnitude lower than that of the HfO_x_/AlO_y_ (15:5) SLL device and the *V*_F_ drops to 1.25 V. The resistance state can be tuned gradually by regulating the *I_CC_* during the SET operation and the stop voltage in the RESET process. As illustrated in Figure 3c,d, the SET process can have up to 160 levels of resistance state while the RESET process can have up to 62 levels of resistance state. These findings demonstrate the HfO_x_/AlO_y_ SLL memristor's outstanding analog resistance switching characteristic, which meets design objectives and indicates a high potential for neuromorphic computing applications.

**Figure 4:**
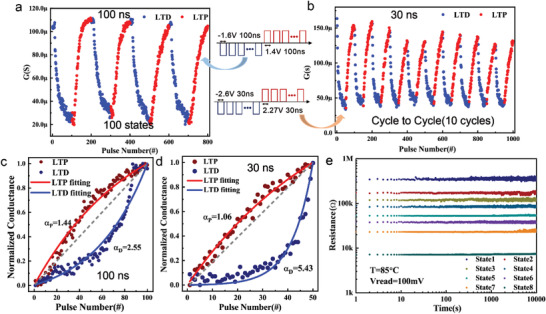
Synaptic characteristics of the HfO_x_/AlO_y_ SLL memristor. a) Conductance update of the HfO_x_/AlO_y_ SLL memristor by applying identical pulses. The device was programmed by 100 depression pulses of ‐1.6 V (100 ns) for the long‐term depression (LTD) process and 100 potentiation pulses of 1.4 V (100 ns) for the long‐term potentiation (LTP) process. The sequence was repeated four times and the conductance can be tuned from 20 to 100 µS. b) Conductance update with fast operating speed, the pulse width is 30 ns, the amplitude of potentiation pulses (*P*_P_) is 2.27 V and depression pulses (*P*_D_) is −2.6 V, and the conductance state decrease to 50 levels. c,d) Comparison of the synaptic weight update nonlinearity obtained from the HfO_x_/AlO_y_ SLL memristor with different pulse widths. When the pulse width is 100 ns, *α*_P _= 1.44 and *α*_D_ = 2.55. When the pulse width is reduced to 30 ns, *α*_P_ is improved to 1.06 and *α*_D_ is worsened to 5.43. e) Retention properties for eight conductance levels at 85 ℃.

In the following section, we mimic the synaptic weight update behavior by imposing a series of potentiation pulses (*P*_P_) and depression pulses (*P*_D_). The periodic square wave pulses with the uniform width and amplitude are applied, with no help from methods such as write‐verify or compliance by transistors. This operation approach is in the line with the needs of online training, and have the potential to reduce the area budget of peripheral operating circuits. Initially, for the long‐term depression (LTD) process, 100 continuous depression pulses *P*_D_ of −1.6 V (100 ns) were imposed on the device, and the synaptic conductance fell from 110 to 20 µS. Similarly, for the long‐term potentiation (LTP) procedure, the device was programmed with 100 sequential potentiation pulses of 1.4 V (100 ns) before being SET back to 110 µS. As illustrated in **Figure** **4**a, after four cycles of programming, there are 100 levels of conductance state that can beacquired for the synaptic weight storage. To describe the linearity of weight update during the LTP and LTD processes, the conductance change of LTP (*G*_LTP_) and LTD (*G*_LTD_) with the number of pulses (P) can be modeled by the following equations^[^ ^9^, ^33^ ^]^

(1) $$G_{\mathrm{LTP}}=B\left(1-e^{\left(\frac{-P}{A}\right)}\right)+G_{\min}$$

(2) $$G_{\mathrm{LTD}}=-B\left(1-e^{\left(\frac{P-P_{\max}}{A}\right)}\right)+G_{\max}$$

(3) $$B=\frac{G_{\max}-G_{\min}}{1-e^{\frac{-P_{\max}}{A}}}$$

(4) $$\alpha =\frac{1.726}{A+0.162}$$

where *G*_max_ is the maximum conductance, *G*_min_ denotes the minimum conductance, and *P*_max_ denotes the maximum number of pulses required to tune the device from the minimum to maximum conductance state. *A* is the parameter that determines the weight update's nonlinearity behavior, *B* is simply a function of *A*, and *α* is a parameter characterizing the nonlinearity. The fitting results are demonstrated in Figure 4c; the nonlinearity parameter of HfO_x_/AlO_y_ SLL memristor is 1.44 for LTP and 2.55 for LTD, respectively.

HfO_x_‐based memristors typically have high‐speed switching behavior;^[^ ^13^ ^]^ thus, by sacrificing some of the linearity of LTD and conductance states, the programming speed can be increased further. The programming pulse width can be lowered to 30 ns, the amplitude of *P*_P_ is 2.27 V and the amplitude of *P*_D_ is −2.6 V, in exchange for the conductance state deduction to 50 levels. Figure 4b shows the endurance with 30 ns programming pulses, the conductance change range is expanded from 25 to 150 µS, while cycle to cycle variation of ten cycles is more clearly detected. Figure 4c,d calculate the cost of linearity when two operational techniques are used. Although the latter operational strategy worsens the nonlinearity parameter (*α*_D_) of LTD to 5.43, fortunately, the nonlinearity parameter (*α*_P_) of LTP improves to 1.06, showing virtually full linearity (equal to 1). We examined the critical performance of several major memristive synaptic devices reported recently in Table S2, Supporting Information, and the HfO_x_/AlO_y_ SLL memristor displays the quickest operating speed, while also demonstrating other good performance.

The conductance state retention is also a key reliability indicator for memristive synaptic devices, particularlly for demosntrating inference function.^[^ ^34^ ^]^ The HfO_x_/AlO_y_ SLL synaptic device has outstanding nonvolatile properties, as shown in Figure 4e. For the retention test at 85 ℃, eight conductance states of the device were chosen and monitored by 10 mV read voltage every second. Each state can be held for >10^4^ s without considerable drift. To summarize the benefits of Ti/(HfOx/AlOy)_SLL_/TiN SLL design memristors derived from the simple structure of Ti/HfO_x_/TiN memristor, an analog‐type memristive synapse with excellent linearity of weight update can be obtained, while the great nonvolatile feature and the fastest operating speed of HfOx‐based memristors can be preserved.

### 2.4 Validation in CNN

**Figure 5:**
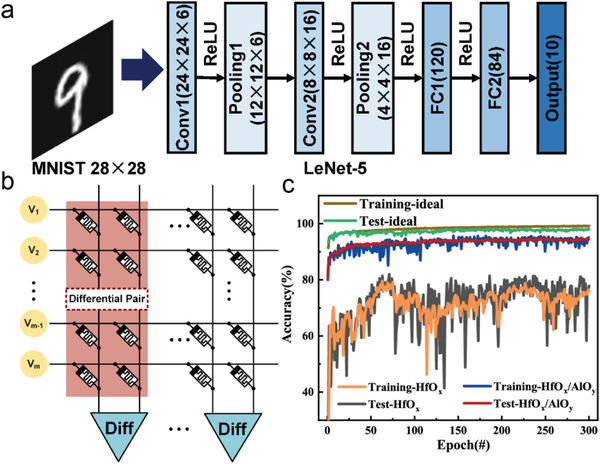
Performance evaluation of the HfO_x_/AlO_y_ SLL memristor and HfO_x_ memristor‐based CNN. a) The LeNet‐5 architecture used in this work for MNIST dataset recognition. b) Illustration of the memristors based convolution kernels. c) The simulated recognition accuracy as a function of the training time.

We constructed a convolutional neural network (CNN) based on memristor crossbar array for the handwritten digit recognition task to evaluate the improvement of HfO_x_/AlO_y_ SLL memristive synapse compared to HfO_x_ memristor in the neuromorphic computing applications. To complete the MNIST benchmark task we use the standard LeNet‐5 structure. **Figure** **5**a depicts the general architecture of LeNet‐5, which consists of two convolutional layers and two average pooling layers, followed by three fully connected layers.^[^ ^35^, ^36^ ^]^ The hardware neural network is implemented by the memristor crossbar array, as shown in Figure 5b, with the weight matrix is represented by the memristor device conductance in the crossbar.^[^ ^37^ ^]^ We employ two memristors connected in parallel as a differential pair, and combining two columns extends the weight to a negative range and takes advantage of greater nonlinearity during LTP. Figure 5c demonstrates the CNN's recognition performance using a HfO_x_/AlO_y_ SLL memristive synapse, a HfO_x_ memristor, and an ideal electronic synapse. The ideal device for the test set has 97.94% handwritten digit recognition, whereas the HfO_x_/AlO_y_ SLL memristive synapse‐based CNN has a 94.95% accuracy, and the HfO_x_ memristor‐based CNN just can achieve 77.8% recognition. Thus, when compared with the HfO_x_ memristor, the HfO_x_/AlO_y_ SLL memristive synapse based CNN improves the handwritten digit recognition by 17.15%, owing mostly to the better linearity provided by SLL design.

## 3 Discussion

**Figure 6:**
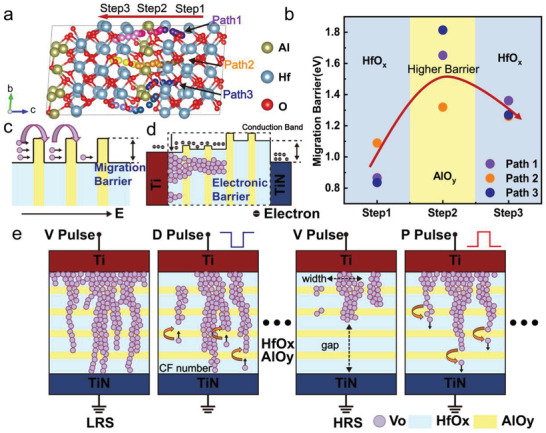
Theoretical explanation of the HfO_x_/AlO_y_ SLL memristor analog switching behavior and the description of the evolution process of CFs. a) Atomic layers model of SLL switching layer with 3 HfO_x_ cycles and 1 AlO_y_ cycle. Three different migration paths of oxygen atoms cross the AlOy layer in HfO_x_/AlO_y_ SLL film, the migration process is divided into 3 steps of HfO_x_‐AlO_y_‐HfO_x_. b) The migration barriers to be overcome for oxygen atoms migrating along different paths in the HfO_x_/AlO_y_ SLL film. The barrier energy is the difference value between the highest energy and the initial energy of each step, the detailed migration energy of each path is shown in Figure S13, Supporting Information. c) The migration process of *V*_O_ under the applied electric field (E) in the HfO_x_/AlO_y_ SLL memristor, where the *V*_O_ needs enough energy to overcome the migration barrier induced by the AlO_y_ layer. d) The relationship between the morphology of *V*_O_ CFs and the energy band in the HfO_x_/AlO_y_ SLL switching layer, described according to the density of electron states of each atomic layer by DFT ‐1/2 calculation^[^ ^43^, ^44^ ^]^ (Figure S15, Supporting Information). e) The evolution process schematics of *V*_O_ CFs during the conductance gradual update process of the HfO_x_/AlO_y_ SLL memristor.

The enhanced synaptic characteristics, derived from the theoretical design of the HfO_x_/AlO_y_ SLL switching layer, can be attributed to the accurate engineering of the HfO_x_ device barrier for conduction path evolution by periodically inserting AlO_y_ barriers slightly higher than the HfO_x_ barrier. We built an atomic layers model of SLL switching layer with three HfO_x_ layers and 1 AlO_y_ layer and used nudged elastic band (NEB) method to calculate the O atom migration barrier,^[^ ^38^ ^]^ which is equivalent to the *V*_O_ migration barrier along different migration pathways. As demonstrated in **Figure** **6**a,b, three different paths to cross the AlO_y_ layer were calculated and the energy barriers required to overcome for O atoms to migrate in AlO_y_ are all 0.06–0.97 eV higher than those required in HfO_y_. As a result, Figure 6c,d shows the migration of *V*_O_ and the transform of an energy band in the HfO_x_/AlO_y_ SLL switching layer. Due to its higher barrier, AlO_y_ functions as an impediment on the migration path of *V*_O_, and *V*_O_ progressively overcomes the AlO_y_ barriers to complete the migration, under the intermittently supplied electric field produced by consecutive pulses. The *V*_O_ migration process during SET/RESET in the HfO_x_/AlO_y_ SLL switching layer is similar to hurdling: each spike pulse can only help *V*_O_ pass one or a few AlO_y_ obstacles, and cannot run to the end freely at one stroke. Meanwhile, when CFs develop, the band will be gradually dragged down by the buildup of *V*_O_ defects, which leads to the increase in conductivity. Meanwhile, the migration barrier of O atoms in the SLL layer with thicker AlO_y_ layers was calculated also (described in Figure S14, Supporting Information), the energy required to cross the thicker AlO_y_ layer (≈5 eV) is much higher than that of thinner AlO_y_ layers (1.3–1.8 eV), which reveals the reason that the higher operating voltage of devices with thicker AlO_y_ layers.

Furthermore, the bond energy of Al‐O is higher than that of Hf‐O, according to the results of theoretical calculations (detail is described in Figure S10, Supporting Information) and XPS. Hence, it is more challenging to generate *V*_O_ in AlO_y_ layers, resulting in the formation of multiple‐weak‐filaments rather than a single strong filament.^[^ ^39^ ^]^ As a result of the production of multiple‐weak‐filaments and successful prevention of burst barrier breaking and conduction‐path formation, the evolution of CF becomes a gradual process, leading to the improvement in conductance update linearity. The relaxation of CF is also controlled, thanks to the confinement effect of the Hf‐O‐Al bond on oxygen atoms and the higher *V*_O_ migration barrier of the AlO_y_ layer, making HfO_x_/AlO_y_ SLL memristor have an outstanding data retention capability.^[^ ^40^, ^41^ ^]^ Meanwhile, because the thickness of the AlO_y_ layers is only around an atomic layer, the width of a pulse stimulating the *V*_O_ to cross the barrier does not have to be wide, allowing the memristor to set at a high speed.

The fitting results of the SLL memristor's conduction mechanism during the switching progress likewise significantly support the aforementioned theoretical analysis. Figure 3e and Figure S11, Supporting Information show the fitting results for the low resistance state (LRS), where the conduction of electrons experienced Ohmic conduction, Schottky conduction, and space charge limit current (SCLC) as the applied voltage increased. Compared with the Ohmic conduction of HfO_x_ memristor throughout the LRS as shown in Figure 3f, Schottky conduction and SCLC both indicate that the device does not develop robust CFs in LRS. Furthermore, in the high resistance state (HRS), contrasting the Schottky conduction of HfO_x_ memristor the SLL memristor follows Poole‐Frenkel (PF) emission transport model, indicating that there is some residual CF in the SLL switching layer to assist the electron conduction. We detailed the evolution of the V_O_ CFs during the conductance gradual update process of the HfO_x_/AlO_y_ SLL memristor, based on the findings of theoretical calculation and fits of the conduction mechanism, as shown in Figure 6e. A redox reaction occurred at the interface between the Ti top electrode (TE) and the SLL layer during the forming process, and a portion of Ti TE was oxidized, generating numerous *V*_O_ at the interface. When a forming voltage pulse is applied to TE, *V*_O_ migrates toward bottom electrode (BE) and forms V_O_ CFs between TE and BE, the memristor is set to an LRS. If we then apply a P_D_ to the memristor, *V*_O_ will overcome the migration barrier and migrate toward TE, causing the CFs to rupture and the memristor to reset to a higher resistance state. Because of the blocking effect of AlO_y_ layers, CFs near the TiN electrode is thinner than that near Ti electrode, which would lead to the rupture of CFs primordially taking place at the weaken‐link node around the TiN electrode or AlO_y_ layers during RESET process, different from HfO_x_ memristor. Meanwhile, a single *P*_D_ cannot move the *V*_O_ very far, and CFs can only be partially broken. A series of *P*_D_ will cause the *V*_O_ to constantly overcome the AlO_y_ barrier and migrate to TE, the CFs will be more fully broken and the filament gap with BE will become wider and wider until the memristor is reset to HRS. Similarly, a series of *P*_P_ will assist *V*_O_ in repeatedly breaking through the AlO_y_ barrier and migrating to BE, resulting in the gradual growth of CFs and the gradual improvement of conductance until the memristor is set to LRS again. When compared with a robust CF in a HfO_x_ memristor, which can provide a larger conductance range for modulation, three elements of CF in the SLL memristor can be regulated: number, width, and gap.

## 4 Conclusion

In conclusion, by upgrading the simple sandwich structure to a superlattice‐like structure we proposed and validate a new theoretical design idea of switching layer for a memristive synaptic device with improved memristive synaptic properties. The form and break processes of CFs can be adjusted from abrupt to gradual by leveraging the varied migration barriers of *V*_O_ in alternating SLL layers to optimize the control of the CFs, thereby accomplishing analog resistance switching. To realize the SLL switching layer we use HfO_x_ and AlO_y_ films and show a Ti/(HfO_x_/AlO_y_)_SLL_/TiN memristor. According to the various optimization results, reducing the thickness of AlO_y_ layers can decrease the V_F,_ and increasing the number of cycles can increase the number of conductance states, both of which are compatible with our findings. The memristor exhibits the best analog switching performance with 160 levels of resistance state for the SET process and 62 levels of resistance state for the RESET process when the SLL switching layer is constructed of 13 cycles of three atomic layers of HfO_x_ and one atomic layer AlO_y_. The SLL memristor offers synaptic performance, with linear conductance update of a linearity parameter up to 1.06, 100 levels of conductance state, outstanding operating speed (30 ns), data retention (85 ℃, 10^4^ s), scalability, and CMOS process compatibility. Meanwhile, CNN based on the SLL memristive synapse boosts the handwritten digit recognition accuracy to 94.95%. Finally, using NEB calculations and fitting the conduction mechanism, the physical nature of the analog switching is explored and the formation and break process of CFs is described.

## 5 Experimental Section

### Device Fabrication

The HfO_x_/AlO_y_ SLL memristor and HfO_x_ memristor were fabricated as follows. First, a 100 nm TiN BE layer was deposited on a SiO_2_/Si substrate by DC sputtering. Second, HfO_x_/AlO_y_ SLL layer or HfO_x_ layer was deposited by atomic layer deposition (ALD, Beneq TFS200) at 250 ℃, using H_2_O, TEMA‐Hf, and TMA as precursors. Particularly, for the HfO_x_/AlO_y_ SLL layer, HfO_x_ and AlO_y_ were alternatively deposited in atomic layer form, first deposit 3 atomic layers HfO_x_, then deposit one atomic layer AlO_y_, and repeating the above steps for 13 cycles. Finally, 50 nm Ti TE layer and 10 nm Pt capping layer were all deposited by DC sputtering. TE and capping layer were patterned, and the cell area is 100 µm×100 µm.

### Electrical Property Measurement and Device Characterization

All the electrical measurements were conducted with a Keysight B1500A connected with a Cascade MPS150 probe station in the air. Cross‐section TEM specimens of the memristor devices were prepared using an FEI Helios 450s dual beam focused ion beam system by a Ga ion beam with 30 keV energy. The final thinning and cleaning process was finished at 5 and 2 keV. The HRTEM and energy dispersive X‐ray spectroscopy (EDS) imaging were conducted at 200 kV on an FEI Titan Themis 200 microscope equipped with a spherical aberration corrector and a Bruker Super‐X EDX system. XPS spectra were obtained with AXIS‐ULTRA DLD‐600W equipment to determine chemical binding states and atomic ratio. The binding energy was calibrated with the position of the C1s peak at 285 eV.

### Ab Initio Calculations

DFT calculations used the plane‐wave based VASP. Besides, the generalized gradient approximation (GGA) was used for the exchange‐correlation energy, within the Perdew–Burke–Ernzerhof (PBE) functional.^[^ ^42^ ^]^ A constant 500 eV plane‐wave kinetic energy cutoff was used throughout the calculations. The valence electron configurations are 5p, 5d, and 6s for Hf, 2s and 2p for O, 3s, and 3p for Al. The amorphous model contains 306 atoms with about 15 Å for three sides. An atomic layers model of SLL switching layer (3 HfO_x_ layers and 1 AlO_y_ layer) with at least 185 atoms was set up to simulate the migration difficulty of O. For the atomic layers model, an equal‐spacing 2 × 2 × 1 k‐mesh was used for Brillouin zone sampling, while another 2 × 2 × 2 mesh was used for amorphous model. Migration barriers were calculated using a climbing image nudged elastic band (CI‐NEB) method,^[^ ^38^ ^]^ and the migration process is divided into three steps of HfO_x_‐AlO_y_‐HfO_x_ for each path.

### Statistical Analysis

To ensure the representative of the sample data, statistical tests were performed and the statistical results were presented in Supporting Information. First, the device‐to‐device variation of basic resistive switching behavior was analyzed, resistance data in Figure S16, Supporting Information were obtained by switching 15 independent HfO_x_/AlO_y_ SLL memristor cells, and each cell was operated for ten cycles. The mean value of LRS is 2.6 kΩ and of HRS is 59.2 kΩ, the standard deviation of LRS (*σ*_LRS_) is 1.1 kΩ and *σ*_HRS_ is 7.8 kΩ. Figure S17, Supporting Information shows the DC characteristics of five HfO_x_/AlO_y_ SLL memristor cells operated for ten cycles, all the cells exhibited bidirectional analog switching behavior. Then, the device‐to‐device variation of conductance update was counted in Figure S18, Supporting Information, where the data of LTD and LTP process were acquired from five samples with the 30 ns operating pulse. The mean coefficient of variation of LTD (CV_LTD_) is 8.38% and the mean CV_LTP_ is 12.08%, which were from calculating the CV of conductance corresponding to each pulse and averaging. Meanwhile, the cycle‐to‐cycle variation of HfO_x_/AlO_y_ SLL memristor's conductance update was analyzed also, the mean CV_LTD_ and CV_LTP_ of the data with 100 ns operating pulse (Figure 4a) are 10.51% and 11.53% respectively, and for the conductance update with 30ns operating pulse (Figure 4b), the mean CV_LTD_ is 9.68% and CV_LTP_ is 7.68%.

## Conflict of Interest

The authors declare no conflict of interest.

## Author Contributions

C.W. and G.‐Q.M. contributed equally to this work. C.W., X.W., and X.M. proposed the design of superlattice‐like memristor. C.W., M.H., Z.Z., and W.C. contributed to the device fabrication. C.W. performed the device measurement with guidance from X.W. G.M. performed the ab initial calculation with guidance from J.Y. and K.X.. E.H. carried out the neural network simulation with guidance from X.W. C.W. and X.W. wrote the paper. K.X. and X.M. provided suggestions for the project. All authors discussed the results and commented on the manuscript. X.W. and X.M. supervised the project.

## Supporting information

Supporting Information

## Data Availability

The data that support the findings of this study are available in the supplementary material of this article.
