# Supplementary material for: HfOx/AlOy Superlattice‐Like Memristive Synapse
Source: Adv Sci (Weinh). 2022 May 29;9(21):2201446. doi: 10.1002/advs.202201446 (PMC9313512; doi:10.1002/advs.202201446)
Supplement: Supplementary file 1 — Supporting Information [file ADVS-9-2201446-s001.pdf]

Supporting Information

HfOx/AlOy Superlattice-Like Memristive Synapse

Chengxu Wang, Ge-Qi Mao, Menghua Huang, Enming Huang, Zichong Zhang, Junhui Yuan, Weiming Cheng, Kan-Hao Xue, Xingsheng Wang, Xiangshui Miao*

1. The binary switching behavior of HfO_x-based memristor

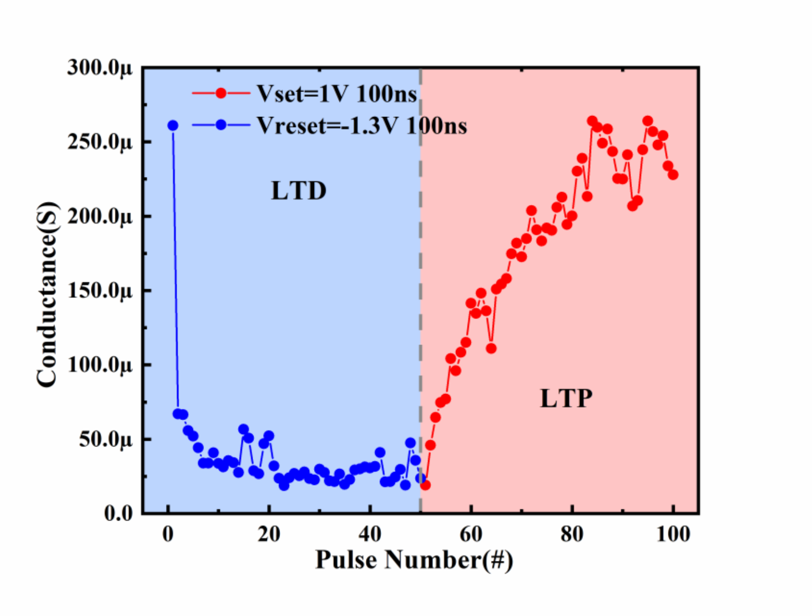

Figure S1. Long-term synaptic plasticity of Ti/HfO_x/TiN memristor. The device was programmed by 50 depression pulses of -1.3V (100ns) for the LTD process and 50 potentiation pulses of 1.4V (100ns) for the LTP process. An abrupt switch occurred during the LTD process.

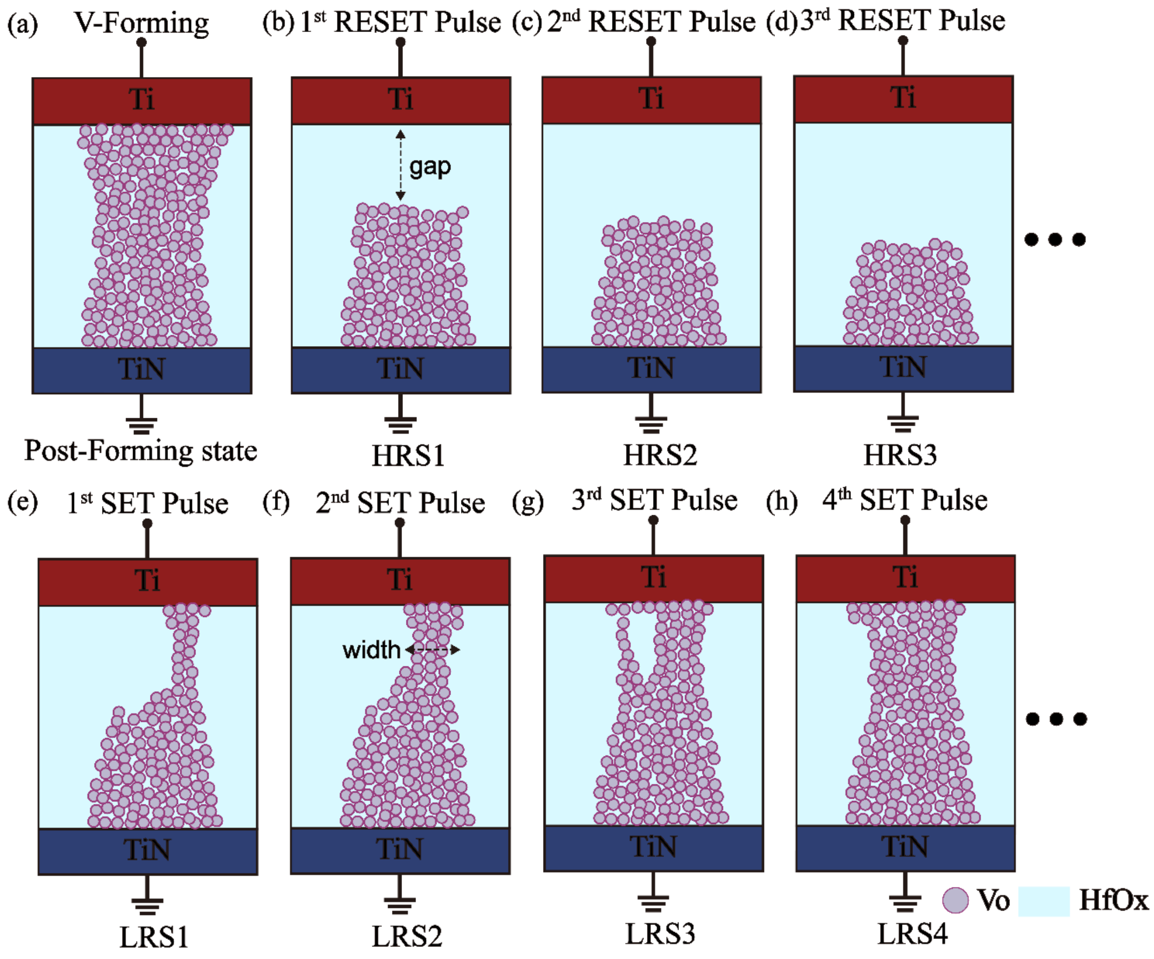

Figure S2. Schematic illustration of the dynamic evolution of V_O CFs for Ti/HfO_x/TiN memristor under the continuous programming pulses. a) Post-Forming state, there is a robust CF formed during the forming process. b) There is a large gap formed between the top electrode (TE) and CF after the application of the first RESET pulse, which is corresponding to the abrupt decrease of conductance with the first RESET pulse applied as shown in Figure S1. c) d) Once the large gap formed, the following RESET pulses are hard to enlarge it and the decrease of conductance will be slower. Thus, the linearity of LTD for the Ti/HfO_x/TiN memristor is non-ideal. For the LTP process, e) after the first SET pulse is applied, a weak CF will form between the residual robust CF and TE and the conductance will increase gradually. f) g) h) With the subsequent series of SET pulses applied, the width of the weak CF will increase until becomes a strong CF again and the conductance will increase gradually at the same time.

2. Calculation of V_O formation energy

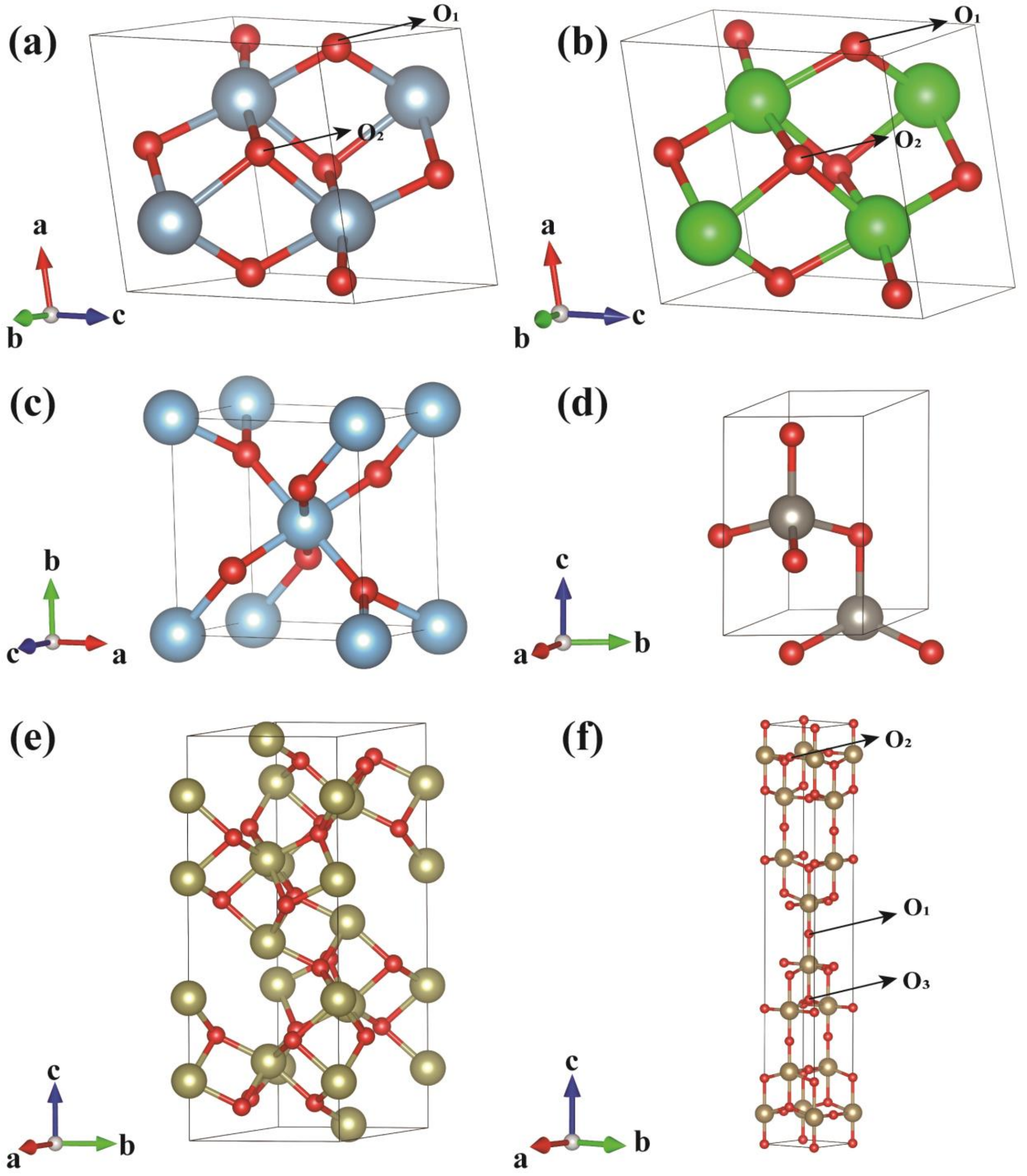

Figure S3. Primitive cell structures of 6 common binary metal oxide memristive materials. a) Structure of m-HfO_2. b) Structure of m-ZrO_2. c) Structure of $P4_2/mnm$-TiO_2. d) Structure of $P6_3mc$-ZnO. e) Structure of $R\bar{3}c$-Al_2O_3. f) Structure of γ-phase Ta_2O_5.

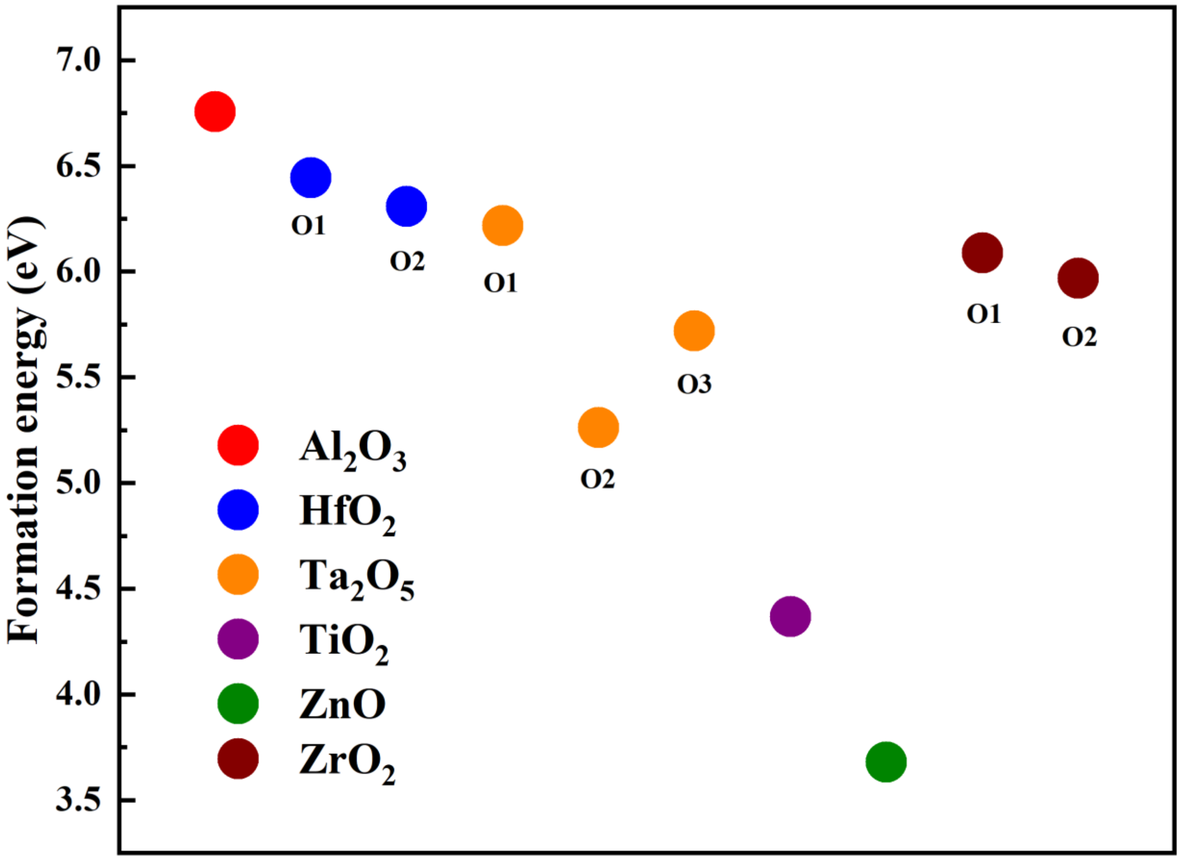

Figure S4. V_O formation energy of 6 binary metal oxide memristive materials, where HfO_2, Ta_2O_5 and ZrO_2 own oxygen vacancies with different positions.

To screen out the suitable combination of materials for SLL switching layer, the V_O formation energies of 6 common binary metal oxide memristive materials were calculated, including m-HfO_2, m-ZrO_2, TiO_2, ZnO, Al_2O_3, and γ-phase Ta_2O_5, as described in **Figure S3**. The V_O formation energy represented the difficulty of generating V_O in the oxide materials, which can partly reflect the difficulty of V_O migration. The V_O formation energy was calculated by the Vienna Ab initio Simulation Package (VASP), where the generalized gradient approximation (GGA) was used for the exchange-correlation energy, within the Perdew–Burke–Ernzerhof (PBE) functional. For each structure, a supercell of at least 10Å×10Å×10Å was established for the calculation of V_O formation energy. A constant 500 eV plane-wave kinetic energy cutoff was used throughout the calculations. The valence electron configurations are 5p, 5d and 6s for Hf and Ta, 3s and 3p for Al, 3p, 3d and 4s for Ti, 3p and 3d for Zn, 4s, 4p, 5d and 5s for Zr, 2s and 2p for O. In the self-consistent calculation, the convergence precision

of the total system energy is less than 10^{-6} eV/Å. The V_O formation energy is calculated from equation S1:

$$E_f = (E_v - E_p) + \mu_O \qquad \text{(S1)}$$

where E_f represents the formation energy of V_O, Ev is the energy of a supercell with an V_O, Ep is the energy of a supercell without V_O, and μ_O is the chemical potential of oxygen atom (-4.96 eV).[1]

The calculated results are presented in **Figure S4**, where $E_f(Al_2O_3)>E_f(HfO_2)>E_f(ZrO_2)>E_f(Ta_2O_5)>E_f(TiO_2)>E_f(ZnO)$, consistent with the previous reports.[2, 3] Hence, if we choose HfO_2 as the basic resistive switching material, only Al_2O_3 can be used as the barrier layer. According to the calculated results, Al_2O_3 is suitable to be used as a barrier layer for its highest E_f, and the blocking effect would be more significant when combined with the materials with lower E_f such as TiO_2 or ZnO.

3. Composition Optimization of HfO_x/AlO_y SLL film

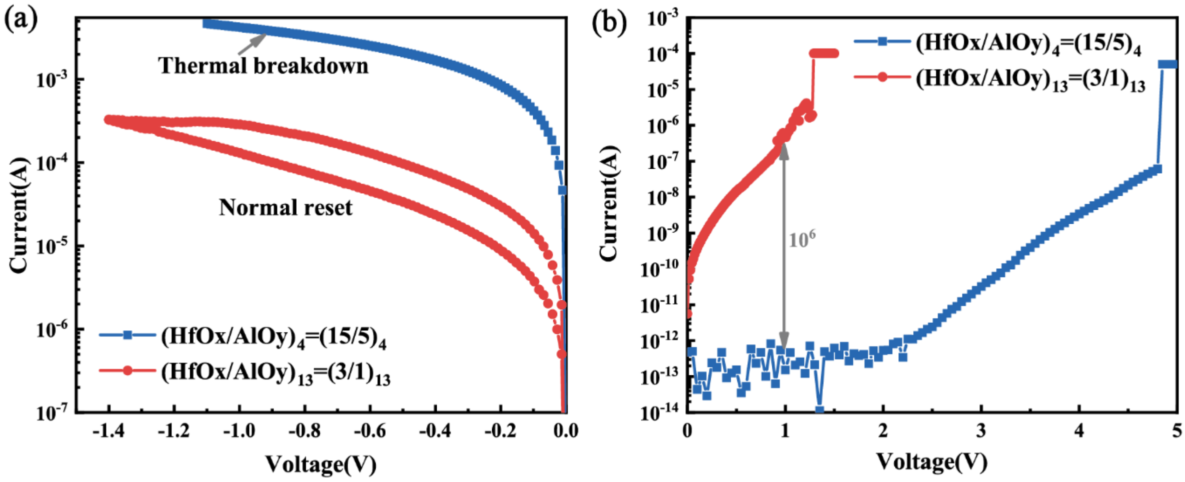

Figure S5. Forming process comparison of thick SLL memristor (4 cycles of 15 atomic layers of HfO_x and 5 atomic layers AlO_y) and thin SLL memristor (13 cycles of 3 atomic layers of HfO_x and 1 atomic layer AlO_y). a) Post-forming state of two memristors, the thick SLL memristor is prone to thermal breakdown during the forming process. b) Forming process comparison, the initial resistance of thick SLL memristor is 6 orders of magnitude higher than that of thin SLL memristor, in addition, the forming voltage of thick SLL memristor is about 4.8V and that of thin SLL memristor is 1.25V.

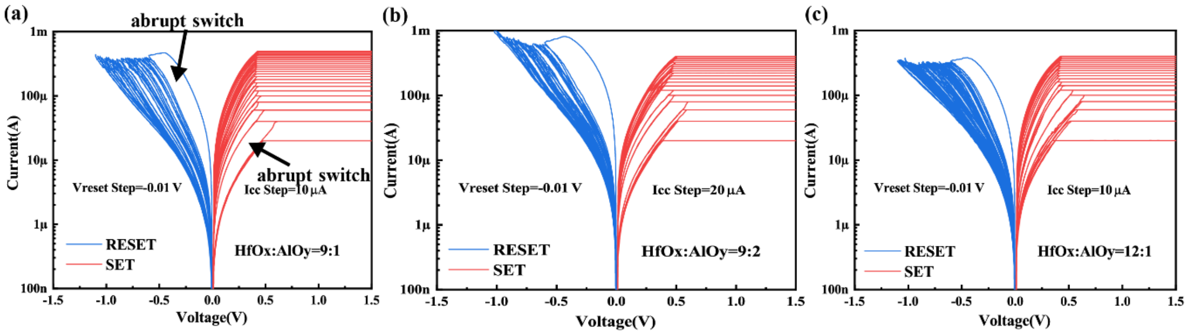

Figure S6. Multi-level resistance comparison of SLL memristor with different HfO_x/AlO_y SLL film compositions. a) SLL device with 5 cycles of 9 atomic layers of HfO_x and 1 atomic layer AlO_y, b) 5 cycles of 9 atomic layers of HfO_x and 2 atomic layers AlO_y, c) 4 cycles of 12 atomic layers of HfO_x and 1 atomic layer AlO_y.

According to the optimization results, when the thickness of AlO_y barrier layers is thick, the initial resistance and forming voltage (V_F) of the SLL memristor will become too high, the V_F close to 5V is adverse for the integration of transistors. Meanwhile, the high V_F also increases the possibility of thermal breakdown during the forming process, as shown in **Figure S3**. When the thickness of AlO_y layers decreases to only one atomic layer, the forming voltage will be reduced to 1.25 V and the device is less prone to thermal breakdown. In addition, reducing the thickness of AlO_y layers can decrease the $V_{F,}$ and increasing the number of cycles can improve the number of conductance states, which are consistent with our design expectation. As illustrated in **Figure S4** there are trip points for the SLL memristors with thick HfO_x layers and thin AlO_y layers. When the composition of HfO_x is much higher than that of AlO_y and each AlO_y layer ais just one or two atomic layers, the ability of AlO_y barrier layers to regulate the CFs will be weakened leading to the abrupt switching behavior.

4. Atomic concentration of HfO_x/AlO_y SLL film and HfOx film

Table S1. The atomic concentration of HfO_x/AlO_y SLL film and HfO_x film

Atomic Concentration%	Hf	Al	O	C
$(HfO_x/AlO_y)_{SLL}$/TiN	24.96	15.25	58.12	1.67
HfO_x/TiN	37.53	0	58.97	3.49

X-ray photoelectron spectroscopy (XPS) spectra were obtained with AXIS-ULTRA DLD-600W equipment to determine chemical binding states and atomic ratio of different elements in HfO_x/AlO_y SLL film and HfO_x film. The binding energy was calibrated with the position of the C1s peak at 285 eV. As shown in **Table S1**, the atomic ratio of Hf and Al is about 5:3 in HfO_x/AlO_y SLL film, and both these two films are non-stoichiometric which means there are numbers of V_O formed in the as-deposited film.

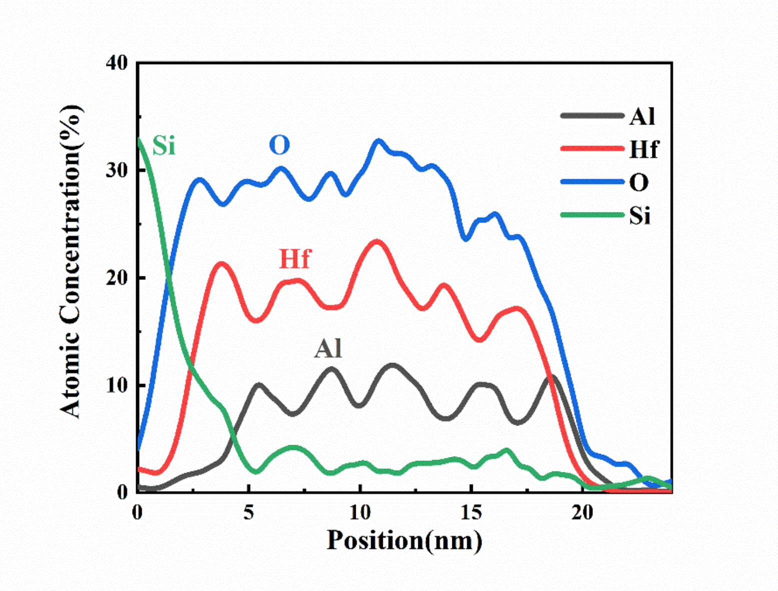

Figure S7. The element profile along the thickness direction of $(30HfO_x/10AlO_y)_{\times 5}$ layer by EDX analysis.

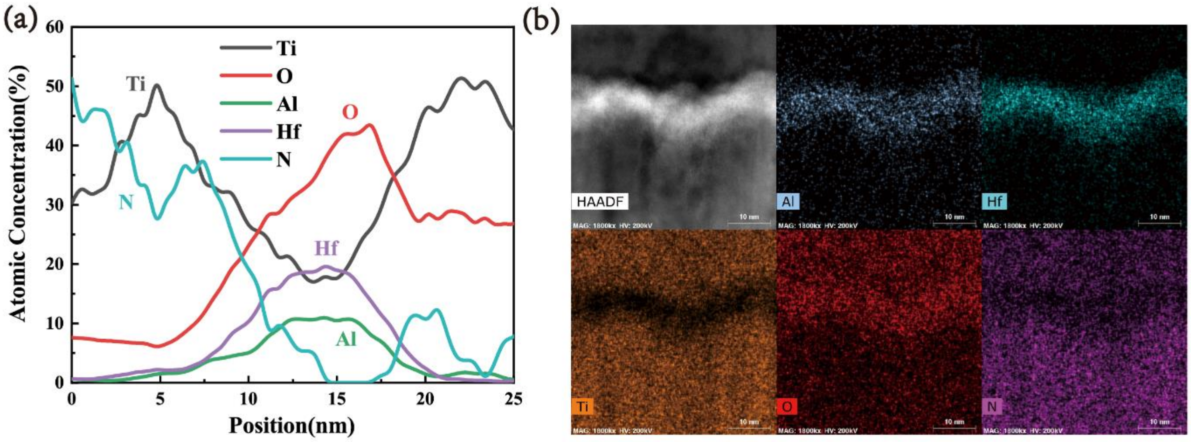

Figure S8. a) The element profile along the thickness direction of the HAO SLL memristor, by energy dispersive X-ray spectroscopy (EDX) analysis. b) EDX mapping of HAO SLL memristor.

EDX line scanning and mapping were employed to check the element distribution in the HfO_x/AlO_y SLL memristor and stratification of HfO_x/AlO_y SLL film, results are shown in **Figure S7, S8**.

5. Operating speed of HfO_x/AlO_y SLL memristor

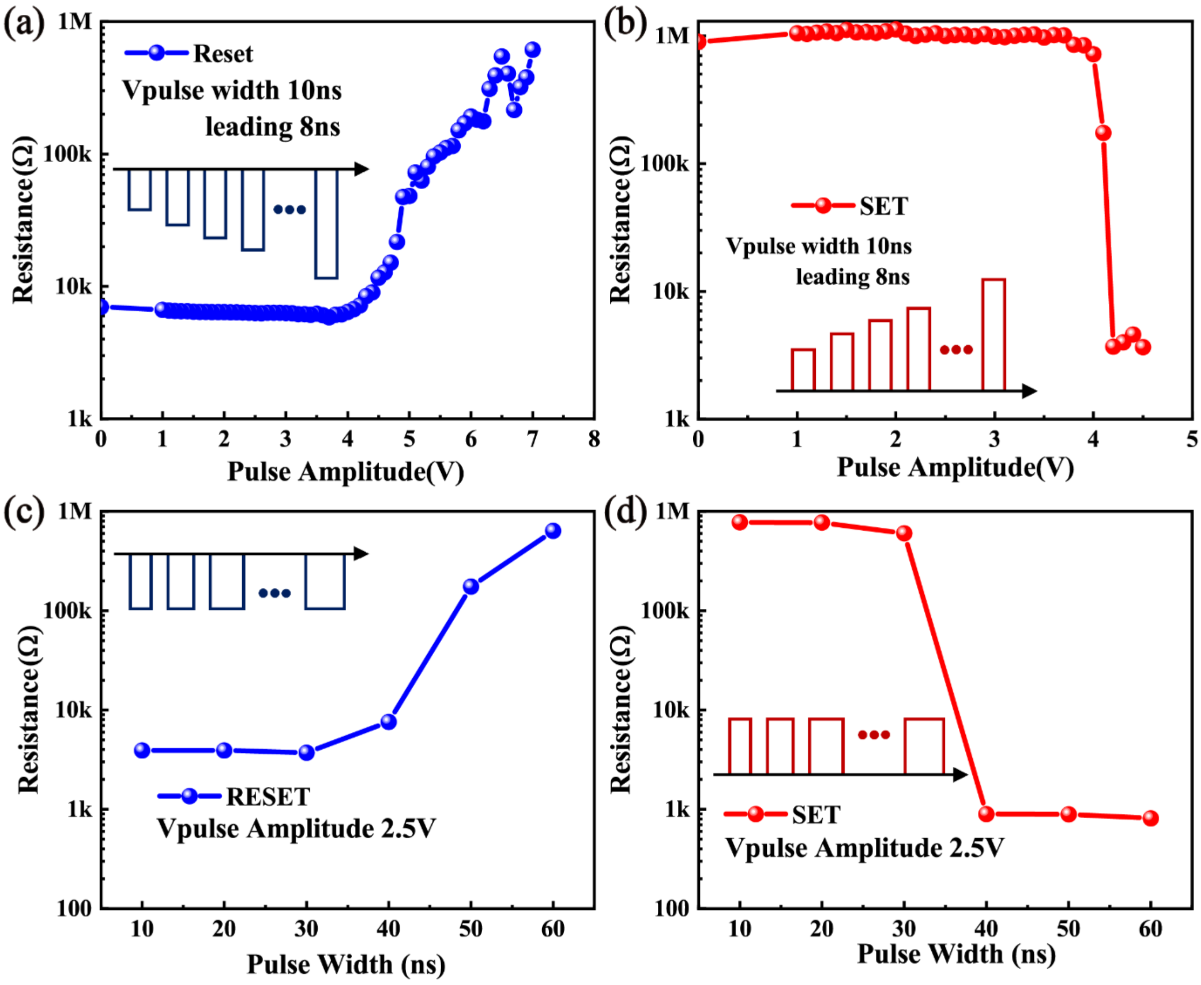

Figure S9. Resistance of HfO_x/AlO_y SLL memristor versus the voltage pulse amplitude and width, a) and c) are reset process, b) and d) are set process. The inset shows the waveform of the applied pulse.

By applying a series of voltage pulses with increasing amplitude or width, we further investigated the response of HfO_x/AlO_y SLL memristor to V pulse. **Figure S7**a, b show the response of resistance to increasing amplitude V_{pulse} with a fixed pulse width of 10ns, which means that the operating speed of the memristor is ≤10ns. **Figure S7**c, d show the response of resistance to increasing width V_{pulse} with a fixed pulse amplitude of 2.5V, which means that the operating power consumption of the memristor is <0.25nJ.

Table S2. Comparison of main performance indicators between this work and memristive synapses with fixed programming pulse

Type of memristive synapses	Program time/spike	LTP nonlinearity	LTD nonlinearity	Retention	Reference
LiSiOx	200ns	1.03	4.58	10^4s@125℃	[4]
PCMO	100μs	5.5	4	N/A	[5]
Ag/GeSe	60μs	N/A	N/A	3×10^3s@25℃	[6]
AlOx/TaOx	10μs	N/A	N/A	10^3s@25℃	[7]
TaOx/Al_2O_3	10μs	2.41	2.77	10^4s@25℃	[8]
TaOx/TiO_2	40ms	0.66	0.69	N/A	[9]
Ag:a-Si	300μs	2.4	4.88	N/A	[10]
OCG-HfOx	100ns	N/A	N/A	10^4s@85℃	[11]
Al:HfO_2	100μs	1.94	0.61	10^3s@120℃	[12]
ETML/HfOx	50ns	0.04	0.63	6×10^2s@125℃	[13]
**HfOx/AlOy SLL**	30ns	1.06	5.43	10^4s@85℃	This work

6. Calculation results of bonding for HfOx/AlOy SLL film

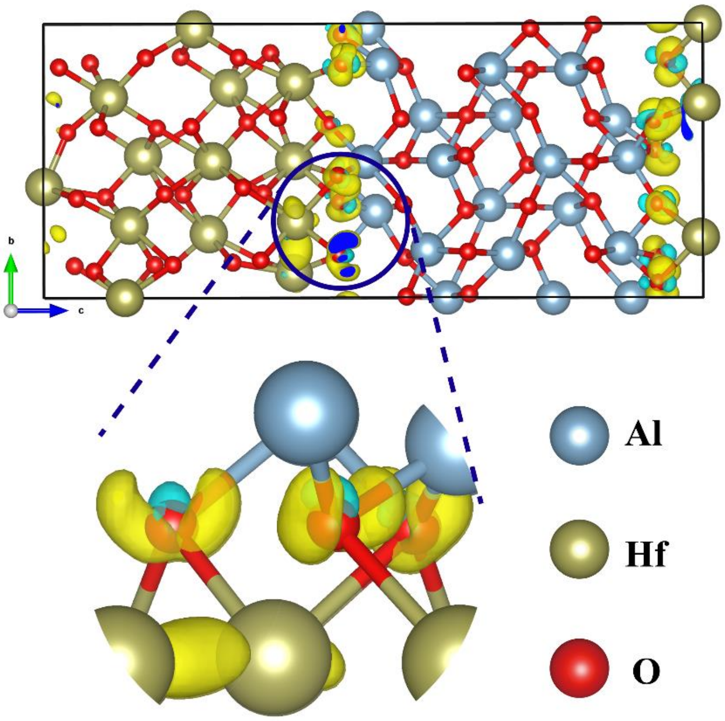

Figure S10. Differential charge density at the interface of the HfO_x layer and AlO_y layer.

To investigate the bonding situation at the interface of the HfO_x layer and AlO_y layer, we established an interface model between HfO_x layer and AlO_y layer and calculated the differential charge density at the interface. **Figure S8** shows the *ab initio* calculation results, compared with Hf atoms, Al atoms at the interface have stronger electron-withdrawing ability and the Al-O bond has higher bond energy than the Hf-O bond, which is consistent with the XPS results as shown in **Figure 2**b,c.

7. Fitting results of conduction mechanism

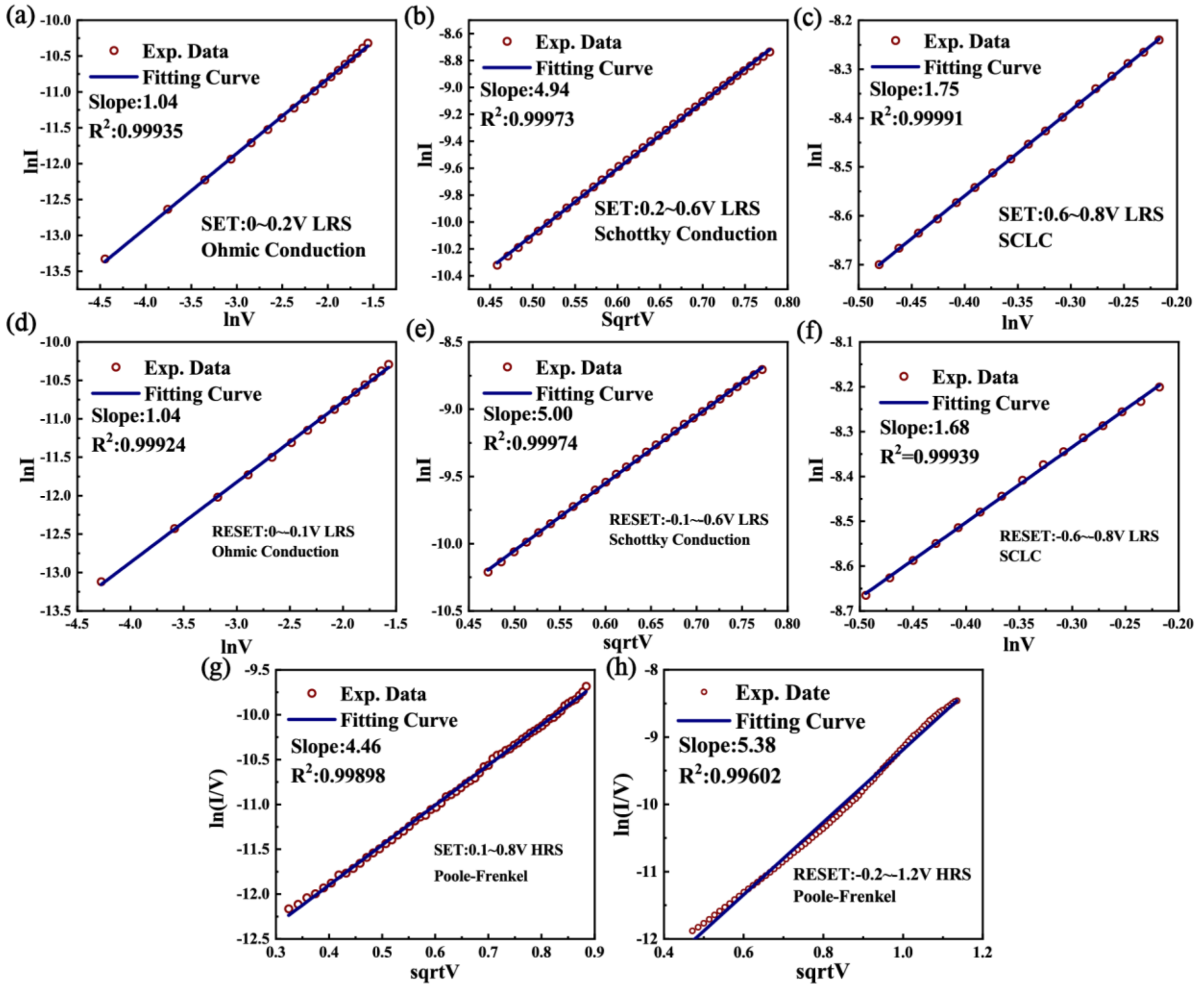

Figure S11. Conduction mechanism fitting results of HfO_x/AlO_y SLL memristor. a) LRS of SET process (0~0.2 V) follows Ohmic conduction. b) LRS of the SET process (0.2~0.6V) follows Schottky conduction. c) LRS of SET process (0.6~0.8 V) follows SCLC. d) LRS of RESET process (0~-0.1 V) follows Ohmic conduction. e) LRS of RESET process (-0.1~-0.6 V) follows Schottky conduction. f) LRS of RESET process (-0.6~-0.8 V) follows SCLC. g) HRS of SET process (0.1~0.8 V) follows Poole-Frenkel emission. h) HRS of RESET process (-0.2~-1.2 V) follows Poole-Frenkel emission.

Fitting expression of conduction mechanisms[14]:

Schottky Emission: $J_{SE} = \alpha T^{3/2} E\mu \left(\frac{m^*}{m_0}\right)^{3/2} exp\left[\frac{-q\left(\phi_B - \sqrt{qE/4\pi\varepsilon}\right)}{kT}\right]$ $\quad \ln I \propto V^{1/2}$ (S2)

Poole-Frenkel Emission: $J_{PF} = q\mu N_C exp\left[\frac{-q\left(\phi_T - \sqrt{qE/\pi\varepsilon}\right)}{kT}\right]$ $\quad \ln(I/V) \propto V^{1/2}$ (S3)

Space-charge-limited Conduction (SCLC): $J_{SCLC} = \frac{9}{8}\varepsilon_I \mu\theta \frac{V^2}{d^3}$ $\quad \ln I \propto \ln V$ (S4)

Ohmic Conduction: $J_{ohmic} = \sigma E = q\mu N_C E exp\left[\frac{-(E_C-E_F)}{kT}\right]$ $\quad \ln I \propto \ln V$ (S5)

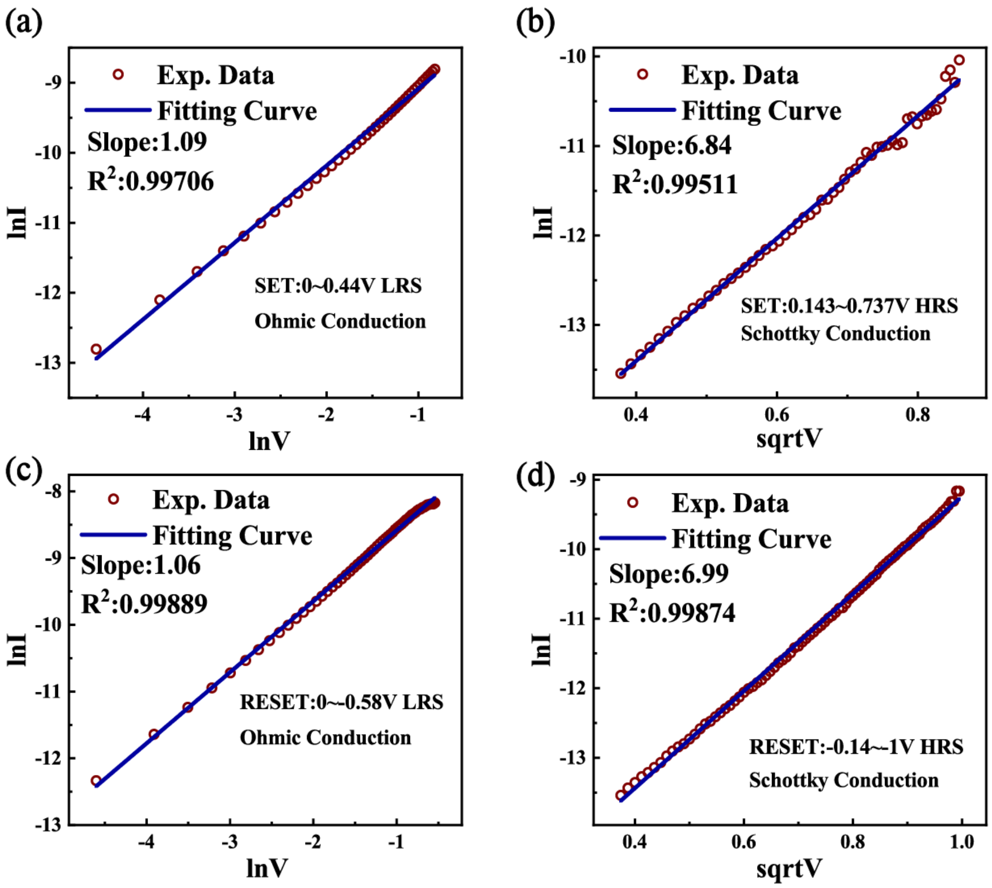

Figure S12. Conduction mechanism fitting results of HfO_x memristor. a) LRS of SET process (0~0.44 V) follows Ohmic conduction. b) HRS of SET process (0.143~0.44 V) follows Schottky conduction. c) LRS of RESET process (0~-0.58 V) follows Ohmic conduction. d) HRS of RESET process (-0.14~-1 V) follows Schottky conduction.

The piecewise fitting results show that, for LRS, there are numerous primary carriers in HfO_x switching layer leading to the Ohmic conduction, indicating the existence of robust CF. When it comes to HRS, the device follows Schottky emission conduction mechanism, which illustrates the drastic rupture of CF during the RESET process.

8. Migration energy of oxygen atoms in HfOx/AlOy SLL film

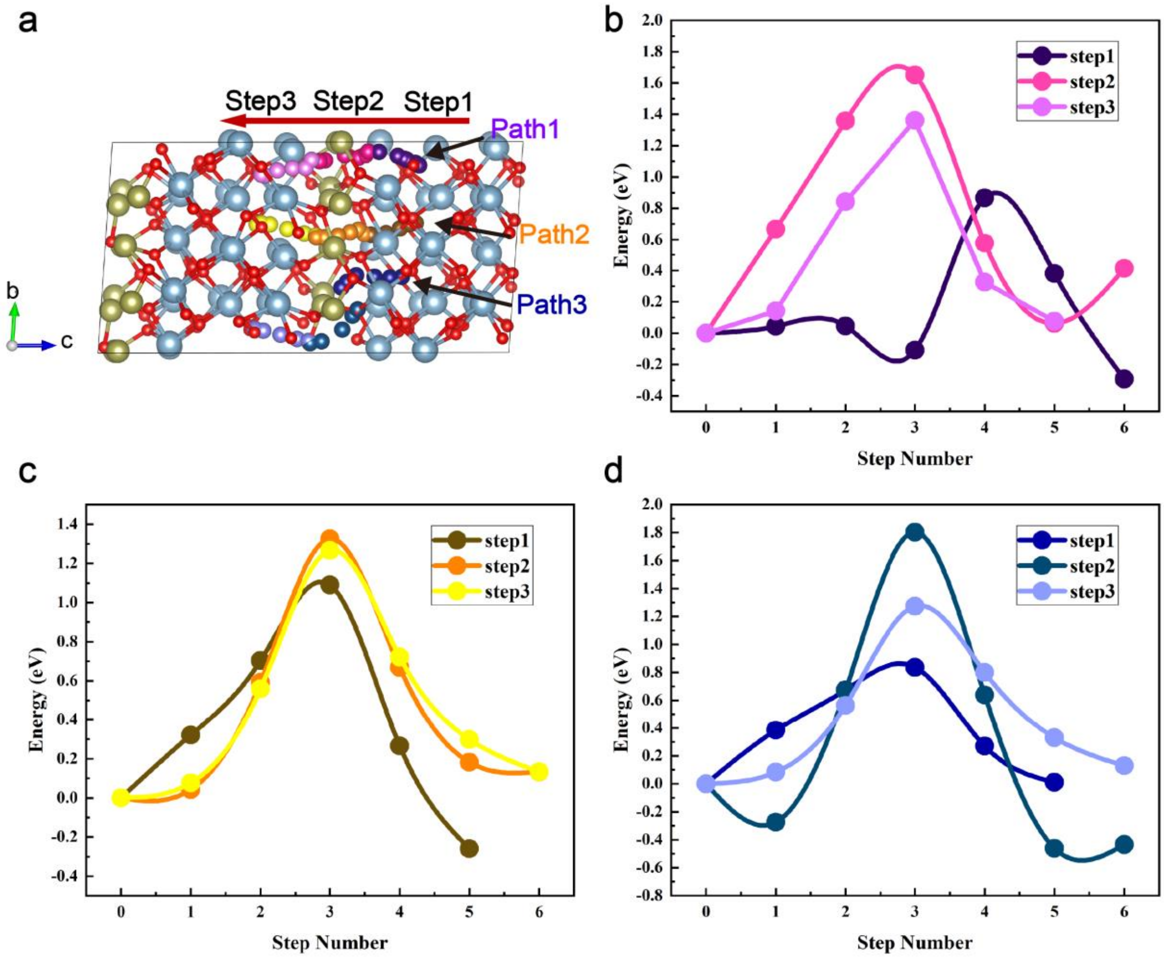

Figure S13. Migration energy of oxygen atoms in HfO_x/AlO_y (3 cycles/1 cycles) SLL film. a) Atomic layers model of SLL switching layer with 3 HfO_x cycles and 1 AlO_y cycle. 3 different migration paths of oxygen atoms cross the AlO_y layer in HfO_x/AlO_y SLL film, the migration process is divided into 3 steps of HfO_x-AlO_y-HfO_x, and each path is consitituted of 18 points. The migration energy of each step of oxygen atoms migrating along b) path 1, c) path 2, d) path 3. For each path, the migration barrier of oxygen atoms cross the the AlO_y layer is higher than that of the HfO_x layers.

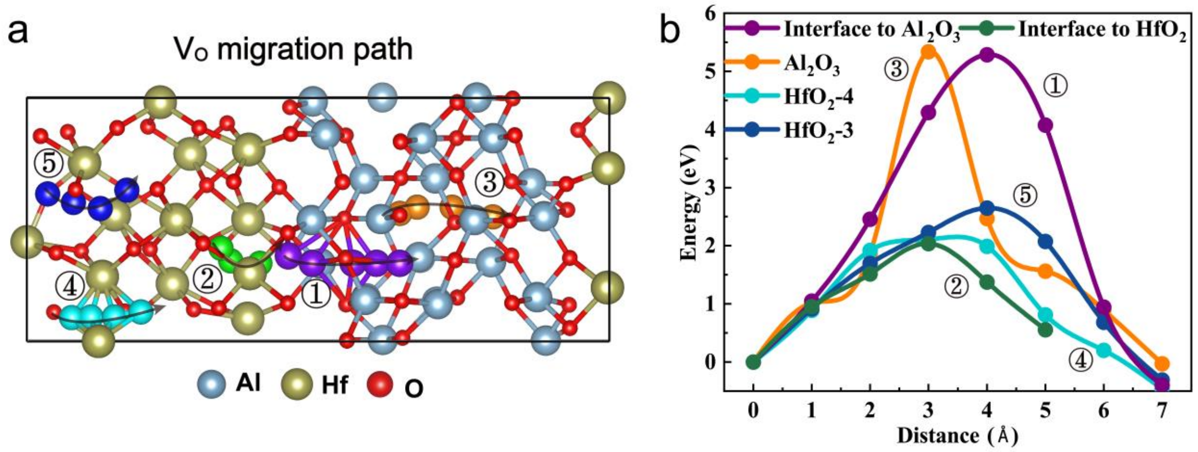

Figure S14. Migration energy of oxygen atoms in HfO_x/AlO_y (3 cycles/3 cycles) SLL film. a) Migration paths of oxygen atom in the HfO_x/AlO_y SLL film. (Path 1 is from interface to Al_2O_3, path 2 is from interface to HfO_2, path 3 is inside the Al_2O_3, path 4 and 5 are inside the HfO_2 with different coordination numbers.) b) The energy barrier to be overcome for oxygen atoms migrating along different paths in the HfO_x/AlO_y SLL film.

Comparing these two SLL models, the migration energy of the film with 1 AlO_y cycle is lower than that of the film with 3 AlO_y cycles, especially for the energy to cross the AlO_y layer. The reduction in migration energy is mainly due to the reduction in the thickness of AlO_y layer, which enlarges the atom spacing of O at the interface because of the interface mismatch. The calculated results of the SLL model with 1 AlO_y cycle demonstrated the blocking effect of AlO_y layer and the speed advantage of the device, in the meantime, the stronger blocking effect of the thicker AlO_y layer illustrated the reason for the higher operating voltage and the deteriorative performance of the corresponding device.

9. Calculation result of DOS for HfOx/AlOy SLL film

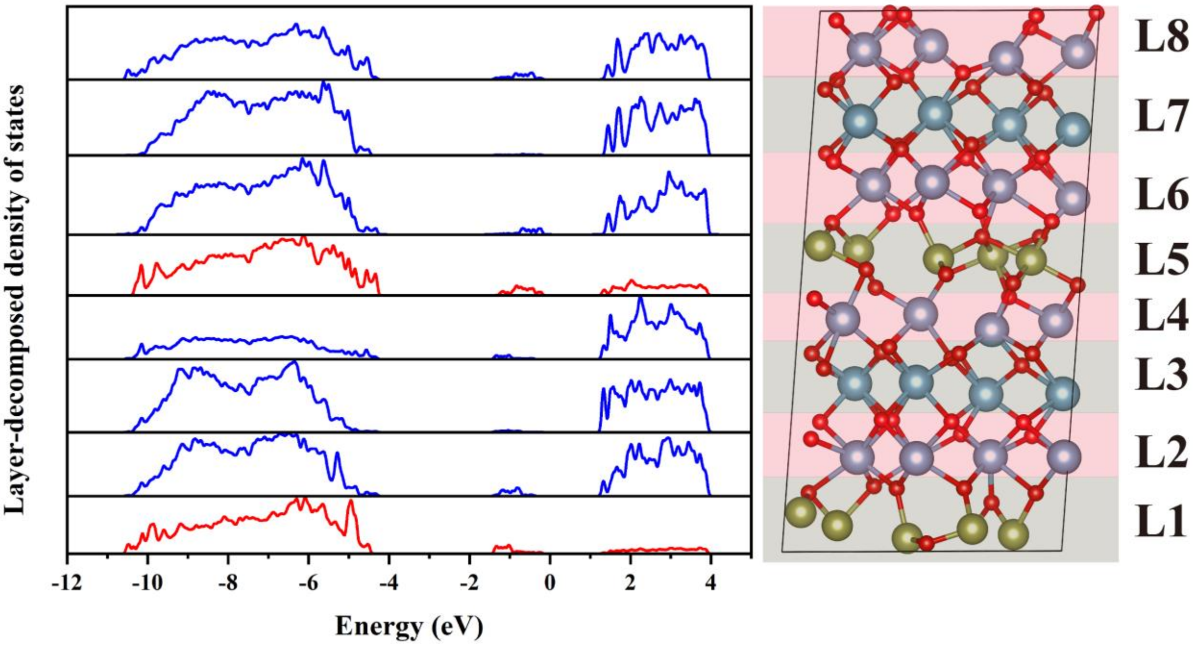

Figure S15. Electronic density of states for HfO_x/AlO_y (3 cycles/1 cycles) SLL film, where the red data is the density of state (DOS) of AlO_y layer and the blue data is the DOS of HfO_x layer. The abnormally tiny DOS for the "conduction band" at the AlOy layer actually reflects the impact of adjacent HfOx states, while the hide band gap of AlOy serves as a barrier for electron conduction.

10. Statistical analysis of device-to-device variation for HfO_x/AlO_y SLL memristor

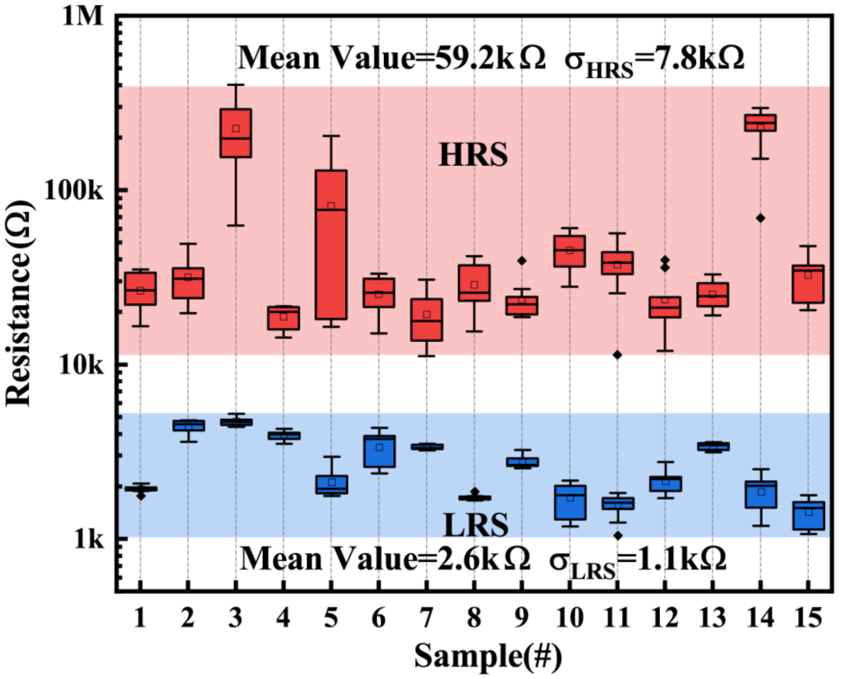

Figure S16. Resistance statistical results of 15 independent HfO_x/AlO_y SLL memristor samples, and each cell was operated for 10 cycles. The mean value of LRS is 2.6 kΩ and of HRS is 59.2 kΩ, the standard deviation of LRS (σ_{LRS}) is 1.1 kΩ and σ_{HRS} is 7.8 kΩ. Due to the analog switching behavior, the distribution of resistance state is uneven.

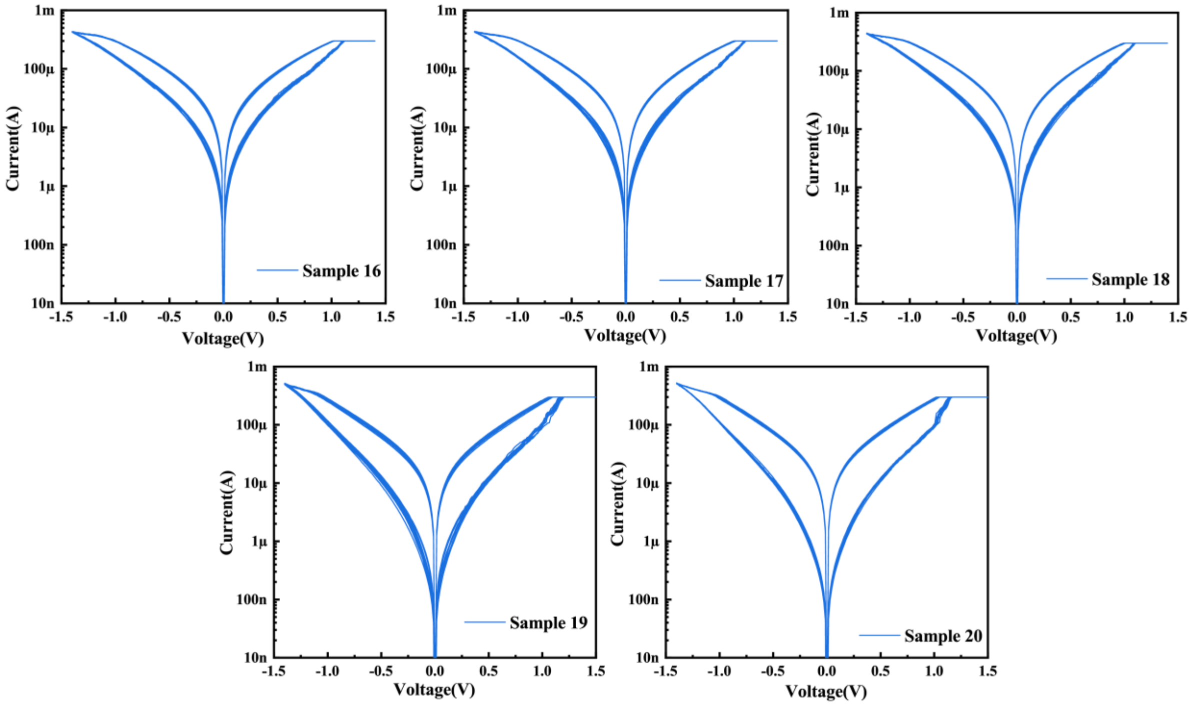

Figure S17. DC characteristics of 5 HfO_x/AlO_y SLL memristor samples operated for 10 cycles, all the cells exhibited bidirectional analog switching behavior.

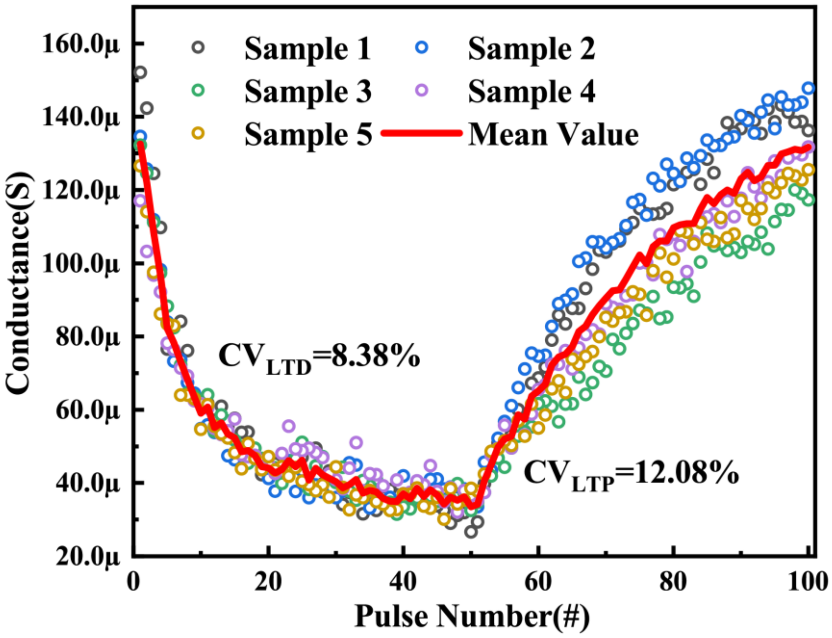

Figure S18. The device-to-device variation of HfO_x/AlO_y SLL memristor's conductance update, where the data of LTD and LTP process were acquired from 5 samples with the 30ns operating pulse. The mean coefficient of variation of LTD (CV_{LTD}) is 8.38% and the mean CV_{LTP} is 12.08%, which were from calculating the CV of conductance corresponding to each pulse and averaging.

Reference

[S1]J. H. Yuan, L. H. Li, W. Zhang, K. H. Xue, C. Wang, J. Wang, X. S. Miao, X. C. Zeng, ACS Appl. Mater. Interfaces **2020**, 12, 13896.

[S2]H. Li, Y. Guo, J. Robertson, *J. Phys. Chem. C* **2015**, 119, 18160.

[S3]Y. Guo, J. Robertson, *Appl. Phys. Lett.* **2014**, 105.

[S4]J. Chen, C.-Y. Lin, Y. Li, C. Qin, K. Lu, J.-M. Wang, C.-K. Chen, Y.-H. He, T.-C. Chang, S. M. Sze, X.-S. Miao, *IEEE Electron Dev. Lett.* **2019**, 40, 542.

[S5]J.-W. Jang, S. Park, G. W. Burr, H. Hwang, Y.-H. Jeong, *IEEE Electron Dev. Lett.* **2015**, 36, 457.

[S6]Y. Sun, H. Xu, S. Liu, B. Song, H. Liu, Q. Liu, Q. Li, *IEEE Electron Dev. Lett.* **2018**, 39, 492.

[S7]Y. Sun, H. Xu, C. Wang, B. Song, H. Liu, Q. Liu, S. Liu, Q. Li, *IEEE Electron Dev. Lett.* **2018**, 39, 1298.

[S8]P.-Y. Jung, D. Panda, S. Chandrasekaran, S. Rajasekaran, T.-Y. Tseng, *IEEE J. Electron Devices Soc.* **2020**, 8, 110.

[S9]L. Gao, I. T. Wang, P. Y. Chen, S. Vrudhula, J. S. Seo, Y. Cao, T. H. Hou, S. Yu, *Nanotechnology* **2015**, 26, 455204.

[S10] S. H. Jo, T. Chang, I. Ebong, B. B. Bhadviya, P. Mazumder, W. Lu, *Nano Lett.* **2010**, 10, 1297.

[S11] Z. Li, B. Tian, K.-H. Xue, B. Wang, M. Xu, H. Lu, H. Sun, X. Miao, *IEEE Electron Dev. Lett.* **2019**, 40, 1068.

[S12] S. Chandrasekaran, F. M. Simanjuntak, R. Saminathan, D. Panda, T. Y. Tseng, *Nanotechnology* **2019**, 30, 445205.

[S13]W. Wu, H. Wu, B. Gao, P. Yao, X. Zhang, X. Peng, S. Yu, H. Qian, *2018 IEEE Symposium on VLSI Technology*, **2018**, 103.

[S14]E. Lim, R. Ismail, *Electronics* **2015**, 4, 586.
